# Extracts from the Valedictory Address, Delivered at the Commencemence of Session of 1855–6, of Medical Department, Iowa University

**Published:** 1856-04

**Authors:** D. L. M’Gugin

**Affiliations:** Professor of Physiology, Pathology, and Clinical Medicine


### EXTRACTS FROM THE VALEDICTORY, DELIVERED AT THE CLOSE OF THE SESSION OF 1855-56, OF THE MEDICAL DEPARTMENT OF THE IOWA UNIVERSITY.

BY D. L. M'GUGIN, M. D.,
Professor of Physiology, Pathology, and Clinical Medicine.

After referring to the honors paid to medicine in the early ages of the world, and the distinctions conferred upon those who taught and practised it, the speaker proceeded as follows:

"But in this day, among civilized men, when the easel and the pencil, in portraiture, have given place to the streaming bright light of the sun; when the tongue and the pen are found vastly inferior to electricity in the conveyance of thought; when steam has almost annihilated space; when minutes are the measures of distance rather than miles; when science boasts of its great triumphs, and medical science of its numerous and fresh achievements over disease, it is nevertheless slightly regarded and often treated with contumely. Not only so, but the old system of charms, incantations, and witchery, *alias* spiritualism, of the olden times of barbarism and blind superstition, are revived, practiced, and placed superior to it, in efficacy and power. Other heterodox systems of parallel utility and about as reasonable as those which were the concomitants of barbarism and ignorance have sprung up, are *encouraged*, *sustained*, and *practiced*, until death, with its grim and threatening visage, will be recognised approaching, and then safety is sought under the sheltering and protecting ægis of the temples consecrated to old Allopathy, and its powers of protection anxiously invoked, when too often the icy hand of the monster has been already laid upon its victim

I would not here be understood as undervaluing any profession or legitimate calling, but I *do* claim for medicine a more exalted position than is accorded to it, because of the greater and longer protracted labor and study in the acquirement of a competent knowledge of it,—because of the toils, cares, responsibilities, and sacrifices, which by night and by day, are involved in its practice, and—because also of the beneficial results which flow from the efforts of the scientific medical man to community and the world.

Not that I would present his physical toils, his personal sacrifices, or his interminable mental efforts, as the most exacting and crushing, but the super-added responsibilities which are no less important than the preservation of health and life, constituting obligations most sacred and imperative. The fulfilment of such a covenant, imposes a task too great for human accomplishment and constitutes a load beyond human power or endurance. And yet all these sources of mental disquietude and physical discomfort—all the hazards and dangers which daily and hourly beset him, are little known, not often considered, and too lightly estimated. Denied the happiness which springs from the fireside of home, enjoyed by the other professions with little interuption or molestation, debarred the pleasures of social re-unions, always grateful because of the contrast to the scenes he is daily required to witness, he becomes the companion of sorrow, and in his heart every joyful feeling which arises, is, as it were, oxydized and rusts.

> "We are fellows still,
> Serving alike in sorrow. Leaked is our bark,
> And we poor mortals, stand on the dying deck
> Hearing the surges threat."

Even when indulging in the luxury of a stolen hour of enjoyment, he feels that he is basking in a sunshine soon to be obscured by some storm-cloud which will burst in gusts of sorrow and torrents of griefs. And how often has this dread been verified? How often is he dragged, in the midst of temporary enjoyment, away to witness the racking tortures of some sick-stricken fellow being? Instead of the genial bright smiles which gladden the heart, as the bright sun cheers the morning, he sees the tear-drop glistening in the eye or glittering upon the cheek; and the sorrowful expression of the countenance indicates but too surely that a gnawing and envious worm is feeding upon the verdure of the soul. The silvery tones of merry voices are exchanged for the hollow sepulchral cadence of one tending downward to death and the grave, or he is compelled to endure the tortures of the rack itself, when the destroying tyrant steps in and nips, as with a blighting frost, the only bud of promise in the widow's heart, or rudely snaps the only remaining fibre which ties her to life. Now the stern reality of the frailty of our physical organization is thus re-exhibited, and the solemn importance of his painful relation to society, his duties and responsibilities, are re-awakened with impressive force in his mind. Now his moral, his social, and professional obligations, arouse him to the full exercise of his mental capabilities and scientific resources. In this struggle, in this solemn conflict, all past enjoyment is forgotten, and in the awful solemnity of the moment, he is oft inclined to loathe upon himself that he ever dreamed of happiness, in view of the solemn import of his mission. Stung with self-accusation, he vows almost upon the altar of humanity, that he will lead a life of self-denial and monkish exaction.

Wherever he goes, and whatever he does, his mind is engrossed in the abiding thought of those who, prostrated by disease, look anxiously and imploringly upon him for aid and relief for their sufferings. He mingles in the crowd and his heart is sad, while the countenances of others are lit up with smiles. This distracting thought, this perpetual anxiety, this continued grinding care, this alternate hoping and fearing, these scenes of suffering and agony, added to his personal sacrifices, bring melancholy upon the mind and disease upon the body, and hence the average duration of the life of the physician is far under that of either of the other professions.

But alas tor poor human nature, and unhappily for medical science, there are those in the profession, who are dead to the appeals of suffering humanity, too many of a cool, calculating, sordid nature, to whom there is nothing sacred in humanity, and with whom the affinities and consanguineous ties are bonds of a loose, light texture. Such, then, without feeling, are without conscience, and those without conscience, disregard human life. With such monstrous exhibitions of these caricatures of humanity, with such black spots of putrescency upon our membership, it is no matter of surprise that the profession should sink into disesteem and dishonor with the philanthropic and the good.

Medical science, however, is not only a grand scheme of humanity, but it is daring and fearless in the discharge of its attributes and functions. Not only does it inculcate principles of humanity, but it infuses chivalry and valor into the mind. When a malignant epidemic sweeps like a besom of destruction over the land, bearing in its dark and bloody track, death and desolation, when terror inspires the multitude who seek safety in flight, the medical man is found at his post of duty, cheering the living, infusing hope into the minds of the sick, alleviating their sufferings and mitigating the dying pangs of those his skill has failed to save. The gloomy insignia of mourning is every where seen, grief sits darkly upon every countenance, and despair is in every soul. Death meets him at every turn and danger environs him by night and by day,—dangers from an ever present enemy, which, being imponderable and intangible, he cannot conquer or subdue; an enemy which defies forts and fortifications, cannon and guns, sabres and bayonets, balls and shot, conquering but unconquerable, it marches on in solid column, wounding and slaying, and in the midst of this carnage, there stands the physician, binding up the wounded and placing soft pillows under the heads of the dying, and whilst in the discharge of this duty, death may be reaching out his cold hand, and his icy fingers may be then, silently and imperceptibly, clutching upon his own vitals. The secular prints have recorded the noble disinterestedness of medical men, who have rushed to the aid of their suffering fellow men, as the history of the Yellow fever at Norfolk, Portsmouth, and elsewhere, during the past season will show; and also that a number fell martyrs in the cause of humanity. These deserve a monument high as the clouds, and as enduring as brass, on which should be inscribed in bright and unfading letters, their acts of disinterested bravery. The profession should perpetuate their names and their memory so long as the sighing breeze and the moaning winds will sing a sad and solemn requiem to their worth and merits. Nature teaches us to shrink from danger, and the law of self-preservation inculcates the principle, but a noble soul subdues its fears and bravely faces the danger which his nature would incline him to shrink from."

"But this is an example of that moral courage which points to a duty and then it is fearlessly performed, and which, coupled with physical courage, constitutes true greatness.

How inconsistent to laud those who have led an army to battle and left the plain bestrewed with the dead and the dying in their triumphant march to conquest and victory, their deeds of daring trumpeted through the land, every hill is re-echoing and resounding with their praises, and yet these martyrs to humanity "sleep their last sleep," and the world is comparatively silent and indifferent, touching the noble magnanimity of their acts and their noble sacrifice of life itself. If forgotten in this life, a crown awaits them in heaven, brilliant and eternally resplendent."

"The statesman, too, glitters in the sunshine of his fame, and a coronet with brilliants dazzles upon his brow. Even the arrant demagogue, who can lead and mislead, promise and betray, fawn, flatter, and play the sycophant, wear the deceitful show of sincerity upon his face, while there is rottenness and corruption glowing phosphoretically in his treacherous heart. Even he, base as he is, can have his bribed followers in troops at his heels, singing hosannas to his praise. The benefits flowing to society from the efforts of one good and true medical man are of more substantial good to mankind, than the most gifted statesman of the present day, and is deserving of infinitely more honor. We are, however, compelled in candor to admit that there have been those who have, in a most cowardly manner, deserted their posts of duty in times of danger and trial. When this is the case, their position is assigned them in the profession, where they are not regarded with favor by the members, but regarded as indelible stains, and by their acts bringing obloquy upon it.

There is another cause which, I am prepared to admit, is a very serious one, and that is the prevalent opinion that a majority of medical men are infidelic in their views, and from this an inference is drawn that medical science inculcates such doctrines. This, we are prepared to show, is not true. On the contrary, I feel emboldened to declare that he who has applied himself to the cultivation of anatomy alone, until a ripe knowledge is obtained, will not dare ignore the plain, undoubted, and unequivocal proofs of the purposes and designs of an omnipotent hand. The human frame, simple yet complicated in structure, nice but yet wonderful, proclaims in the most clear and pointed manner, the existence of a designer. A court of judicature would not, and could not ask for stronger circumstantial evidence, so convincing as to become almost positive. In addition to this, Chemistry will bring up an array of facts not to be accounted for on any other principle, and physiology gives the confirmatory assurance, constituting it almost an undoubted postulate. If time were allowed me, I would proceed to discuss a few points, in order to place the profession in its true light on this question."

[At this point the speaker proceeded extemporaneously to discuss the arguments which have been presented on both sides of the question, in which he aimed to present briefly, certain physiological arguments in proof of his position.]

"Gentlemen of the graduating class, if you will indulge me in drawing from nautical life a figure, I would now congratulate you upon your safe and *successful* passage through "Hurl Gate Channel," the dangers and difficulties of which, have discouraged and disheartened so many from the attempt. While the land is still in view, the sails still furled, and your inexperienced craft still floating listlessly, awaiting the impress of the sails to give it life, and the helm to direct its course, permit an old weather-beaten tar, after having aided in piloting you through the devious and dangerous straits, to impart a few cautions before he takes final leave.

The quiet surface now so inviting, offering no difficulty, and threatening no danger, woos and invites you onward to a sweep over that surface, *now* reposing as soundly as if in the deep sleep of coma. That calm surface, unruffled by a single breeze, reflects brightly the golden rays of hope in the future. Be not deceived by the glitter which this promise holds out to you, for the slumber which now reposes upon the surface of that vasty deep, finds an analogue in the sleep which precedes the most violent convulsive movements, the result of freshly acquired brain power. The wand of the storm spirit, by a single gesture, from its seat in a tiny cloud, not larger than your hand, can awaken it into the most violent demonstrations of surging, lashing, and foaming. The heavens will grow black, the wind will howl in fury around you, whistle through your cordage, make your sails to flap or rend them into shreds. The waves will dash against the sides of your vessel, your craft may be driven against the cliffs of rocks and become a sad wreck. The calm which will follow, and the bright sunshine will be the more cheering, because of the contrast with the previous overpowering blackness and darkness. Even while enjoying this calm, a new and unexpected danger may threaten you, for a pirate's black hull and bloody banners may contrive to get into your wake and cause you to crowd on all sail and away. And then again you may lose your reckoning, your chart may have been neglected, you may be enveloped in a dense fog, and lose your direction.

Let me again warn you not to be deceived by appearances, nor to be too self-confident. The very first case may be one of unusual perplexity, may demand, for its successful management, the ripest experience, and for its control the most profound science. It is usual with graduates fresh from their *Alma Mater*, to plume themselves upon their ability and capacity to meet and control all the evils of Pandora's box, but you are yet to learn, what others have learned before you, that experience will often teach, what cannot be learned by other means. Too much self-confidence has another evil, it will arrest enquiry and prevent you from keeping pace with medical science, now so progressive. Your books will be forgotten, remain unopened, and become dust-covered. This is sleeping during dog-watch, when a storm is coming athwart the sea. A case presents itself and your prompt and intelligent action is required in order to save life. But you have not kept your reckoning, and you know not what course to pursue. Your want of knowledge has cost a valuable life; a storm is blowing in fury around you, and the sails are flapping angrily against your conscience. Public condemnation is rolling angry waves over you and will overwhelm you. And then again, if successful, your good fortune will be hissed at, and the jealousy of some mean and craven spirit will often prompt to the use of contemptuous epithets, in default of other missives. These are squalls which will soon pass away, and leave you wholly unharmed. Such demonstrations find a fit similarity in the odors which arise from putrescency, or the pirates with dark hulls and red and bloody banners, whose life-blood depends upon the pabulum they derive from the destruction of others.

Be cautious not to throw out too much sail for the dimensions of your vessel, or it will founder. Let your works proclaim your capacity to do good, and a discerning public will note your success. Although this is decidedly a *puffing age*, when persons are found often puffing themselves, or *bribing* its performance, there is nothing more loathsome than the medical demagogue, who has his posters upon every corner to laud him into public favor. If you have not worth, merit, or skill enough to make it manifest, you would find a profitable exercise in application and study, in the culture of virtuous principles, rather than to seek your fame by placing others between you and the world to mirror your greatness. This course often becomes necessary to success, as a transparent body will oppose no obstruction to the light, and therefore no shadow is produced. Putrid bodies become gaseous, and therefore transparent, are soon diffused upon the natural hygienic principle, that pestiferous matters are diluted by diffusion, in order to prevent their deadly effects. Let, then, your own worth and merit constitute a basis for your character, and shun every appearance of charlatanism. Carry no false colors, for they are always meant to deceive and betray public confidence. They are, therefore, the pirate's badge and the freebooter's colors. Apply yourselves diligently to your studies, and let your enquiries and researches go hand in hand with experience. Observe faithfully all phenomena, make yourselves well acquainted with every case which presents itself for your consideration and management, and you will not lose your reckoning, nor will you despair, although surrounded with a dense, dark fog. When the tempest howls, and carries on its wings a dread pestilence, when a victim hangs upon every yard arm, when your decks are strewn with the dead and the dying, and loud moans rend the air, be then calm and resolute, firm and efficient, humane and philanthropic, and you will fulfil the object for which you have started on a stormy and long protracted cruise, which, like that performed by Dr. Kane, will require fortitude, wisdom, and perseverance.

You must preserve your recorded experience for the benefit of those who may follow in your wake, an omission, in the performance of which, will be a breach of the faith you have vowed to keep, and an evidence of your want of interest in the well-being of your profession and of humanity.

Gentlemen, you have nobly struggled for the highest honors in medicine, and you have secured the boon which is most deservedly yours. This well merited honor you have received from the Faculty of the Medical Department of the Iowa University, which has passed its days of trial, and is now in the full tide of prosperity and success. With St. Louis south, and Chicago north, we are most fortunately situated, and without a rival in the State, we occupy our own legitimate geographical position, and neither desire nor wish to interfere with the promising schools in those cities. It gives us much pleasure, when we are assured that all good citizens of the State are gratified with our success, because it is the only integral part of the University now in the successful tide of prosperity.

You have the assurance of the Faculty, that if a faithful and unfaltering discharge of our duties will perpetuate its life and favor its growth, then YOU will have no cause to blush for your Alma Mater, while we trust we shall have no occasion to feel shame because of any taint of dishonor you may bring upon IT.

We are about to separate, and the tie which binds us together in the close relation of teacher and pupil, is soon to be sundered. The world without cannot estimate the nearness of that relation, nor the sacredness of that regard, nor can any one appreciate the deep feeling which oppresses us when time, incorrigible in its demands, and unrelenting in its requirements, bids the hour of separation come. There may be those who look lightly upon the circumstance, but we would prove recreant to our emotional nature, did we separate with feelings of indifference. We know there have been those, who, without a proper affectionate culture, could make even greater sacrifices with cold indifference. These are not less than human monsters, and reflect dishonor upon human nature. Cato divorced his wife and sent her away without an emotion. Brutus surrendered his sons to die, without an expression of sorrow or a tear. With heartless coldness and indifference, Sylla ordered the destruction of six thousand of his own countrymen in one day, and could and did coolly say to the Roman senate, that he was only and simply chastising a few rebels.

The magnitude of *our* duties and responsibilities, and the abiding importance of *your* work, were incidents of grave moment, and eminently calculated to awaken a reciprocal regard. In addition to this, not one single or even slight circumstance has occurred to mar the pleasure of our intercourse, nor have we ever known that an unkind feeling ever once existed among yourselves. The very nature of our relative pursuits were calculated to exalt the mind and urge us to the fulfilment of our great designs and objects. While *we* were proclaiming scientific truths, you were laying up materials with which to fulfil a noble destiny.

That you may prove useful in life, that you may become an honor to your profession, that you may be gratified in all your just and noble aspirations, that you may be prosperous and happy, is the prayer, the earnest heartfelt and anxious prayer, of each member of the Faculty.

